# Vapour pressure deficit control in relation to water transport and water productivity in greenhouse tomato production during summer

**DOI:** 10.1038/srep43461

**Published:** 2017-03-07

**Authors:** Dalong Zhang, Qingjie Du, Zhi Zhang, Xiaocong Jiao, Xiaoming Song, Jianming Li

**Affiliations:** 1 College of Horticulture, Northwest A&F University, Yangling, Shaanxi, China

## Abstract

Although atmospheric vapour pressure deficit (VPD) has been widely recognized as the evaporative driving force for water transport, the potential to reduce plant water consumption and improve water productivity by regulating VPD is highly uncertain. To bridge this gap, water transport in combination with plant productivity was examined in tomato (*Solanum lycopersicum* L.) plants grown under contrasting VPD gradients. The driving force for water transport was substantially reduced in low-VPD treatment, which consequently decreased water loss rate and moderated plant water stress: leaf desiccation, hydraulic limitation and excessive negative water potential were prevented by maintaining water balance. Alleviation in water stress by reducing VPD sustained stomatal function and photosynthesis, with concomitant improvements in biomass and fruit production. From physiological perspectives, suppression of the driving force and water flow rate substantially reduced cumulative transpiration by 19.9%. In accordance with physiological principles, irrigation water use efficiency as criterions of biomass and fruit yield in low-VPD treatment was significantly increased by 36.8% and 39.1%, respectively. The reduction in irrigation was counterbalanced by input of fogging water to some extent. Net water saving can be increased by enabling greater planting densities and improving the evaporative efficiency of the mechanical system.

Sustainable agricultural production is shifting from emphasizing production per unit area towards maximizing the production per unit of water consumed^1^. From a physics perspective, water transport along the soil-plant-atmosphere continuum is a passive process driven by gradients of free energy. The driving force for water movement is determined by the water potential gradient in liquid phase (from soil to leaf) and the difference in vapour pressure deficit (VPD) along the gas phase (from internal leaf to the atmosphere)^2^,^3^. The energetic of water flow along the soil-plant-atmosphere continuum can be optimized by regulating the soil or atmospheric water status. Regulation of the soil water status by irrigation management has long been recognized as an efficient solution for modulating water transport and improving water use efficiency^4^. Under natural conditions, soil moisture varies much less than the leaf-atmosphere flux, which fluctuates in response to a high frequency of atmospheric VPD^5^,^6^. While atmospheric VPD mediates water flow and constrains water productivity to a large extent, the potential to reduce plant water consumption and improve water productivity by regulating VPD is highly uncertain. Regulation of atmospheric evaporative demand to reduce plant water consumption and improve water productivity has received far less attention from growers than traditional methods of irrigation. One possible reason is the difficulties in regulating environmental conditions for field-grown crops.

The increasing sophistication of the greenhouse industry facilitates the regulation of atmospheric evaporative demand. Many mechanical devices and computer programs can be applied to greenhouse atmospheric moisture regulation. Greenhouse operations are moving towards controlling evaporative demand according to VPD because this approach provides direct information about the driving force of transpiration and evaporation^7^,^8^,^9^,^10^. The regulation of VPD has been demonstrated as an efficient solution to simultaneously maintain optimal ranges of temperature and relative humidity and therefore effectively enhance plant photosynthesis and productivity in greenhouse production^11^,^12^. However, the potential to reduce plant water consumption and improve water productivity by regulating VPD is highly uncertain. From physiological perspectives, VPD control has been hypothesized to play an important role in reducing water flow rate and cumulative transpiration by suppressing the excess water driving force.

The aforementioned theoretical hypothesis failed to incorporate water consumption for VPD control as a factor influencing resource investment. In greenhouse production, evaporative cooling approaches such as pad-fan systems and fogging systems are generally the most common and effective strategies for VPD control^13^,^14^. Hence, the regulation of VPD via the evaporative cooling method is challenging under conditions of excessive evaporative demand: although reducing VPD has been hypothesized to suppress the water driving force and reduce plant transpiration, the evaporative cooling system inevitably consumes a certain amount of water. The trade-off between the reduction in transpiration and extra input of fogging water is highly uncertain. Less well documented in the literature is the combination of physiological and system-wide perspective to evaluate the implications of VPD regulation in modulating water transport and water productivity. To bridge this gap, the water transport process (transpired water and input of fogging water) in combination with plant productivity was examined in distinct greenhouse VPD gradients in the present study.

## Results

### Effect of VPD regulation on plant water status

The experimental greenhouses differed in the diurnal variation of micro-environmental factors. On a typical sunny day, high temperature (T > 35 °C) and low humidity were observed in the high-VPD treatment (Supplementary Fig. 1A,B). The average VPD around midday periods (11:00–15:00) decreased from 4.5 KPa to 1.6 KPa in response to humidification (Supplementary Fig. 1C). The midday relative humidity was effectively elevated from 42.5% in the high-VPD area to 61.6% in the low-VPD area. The midday temperature substantially decreased from 38.5 °C in the high-VPD area to 31.3 °C in the low-VPD area. The light intensity showed no significant differences between two compartments (Supplementary Fig. 1D), indicating that fogging had no significant effects on solar radiation transmission.

**Figure 1:**
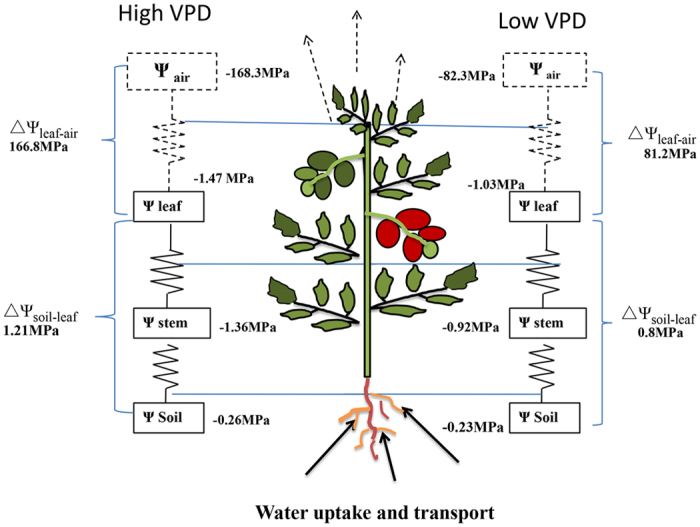
Effect of VPD regulation on the hydraulic driving force for water transport along the soil-plant-atmospheric continuum. The solid and dotted lines represent the series of pathways of water flow in liquid and vapour phase, respectively. ∆Ψ represents the water potential drawdown between the two compartments of the soil-plant-atmospheric continuum.

Regulation of greenhouse VPD by humidification had great effects on plant-water relations. Soil was maintained at a homogeneous water status between two treatments, as indicated by the non-significant difference in predawn Ψ_leaf_ (Table 1). Ψ_air_ reached a maximum negative value at midday, and it was less negative in the low-VPD treatment (Fig. 1). Ψ_leaf_ also experienced a dramatic drawdown in both the low- and high-VPD treatments. This depression in midday Ψ_leaf_ was efficiently moderated by reducing the VPD (Table 1). Taken together, the driving force at the leaf-air boundary was by far the greatest along the soil-plant-atmosphere continuum, which was substantially reduced in the low-VPD treatment (Fig. 1).

Along with the water flow, the whole-plant hydraulic conductance was significantly increased in the low-VPD treatment (Table 1). The diurnal canopy temperature in the two greenhouse compartments followed a similar trend to that of air temperature. The average midday canopy temperature was reduced from 33.7 °C in the high-VPD treatment to 31.3 °C in the low-VPD treatment (Table 1). The plants were less water stressed by reducing VPD according to the value of midday crop water stress index (CWSI) (Table 1). As a result of the plant-atmosphere interaction, reducing VPD effectively moderated leaf desiccation, as indicated by the higher relative water content of the leaves at midday (Table 1).

### Effect of VPD regulation on leaf gas exchange

**Figure 2:**
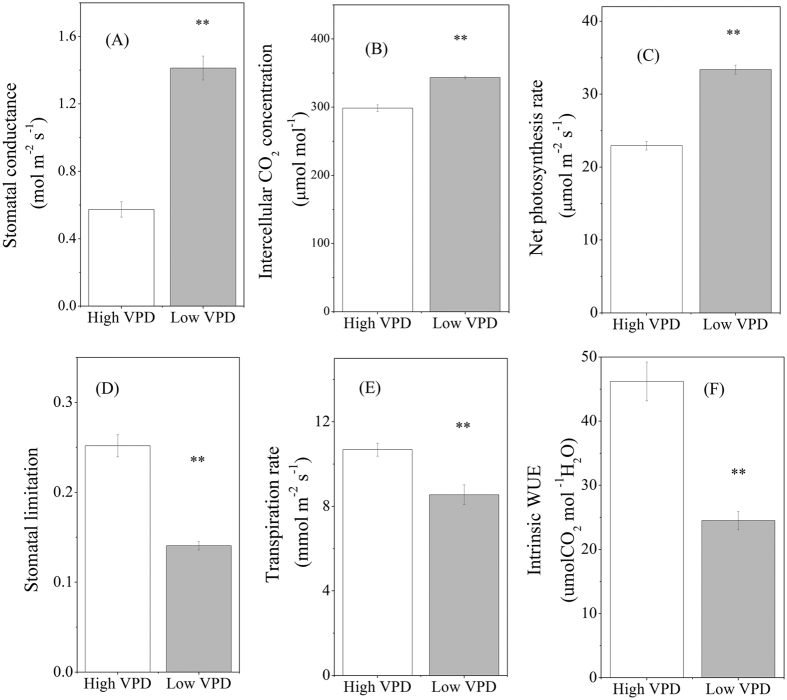
Effect of VPD regulation on leaf gas exchange. The environmental conditions set for the measurements inside the leaf chambers were similar to the growth conditions in the greenhouse. Values are means ± SE (n = 10). Significant differences between the high- and low-VPD treatments were compared using Tukey’s test. **Significant at P < 0.01.

Reducing VPD significantly increased stomatal conductance (Fig. 2A). With the mitigated stomatal limitation for CO_2_ diffusion from the atmosphere to the sub-stomatal cavity, the intercellular CO_2_ concentration and leaf photosynthesis rate increased significantly in the low-VPD-grown plants (Fig. 2B,C,D). Leaf transpiration rate and intrinsic water use efficiency were significantly reduced in the low-VPD treatment (Fig. 2E,F).

### Effect of VPD regulation on plant growth and fruit production

Reducing VPD promoted plant organizational growth: reducing VPD caused a substantial increase in leaf length, with correspondingly faster leaf elongation rates (Supplementary Table S1). Reducing the VPD also significantly increased the stem diameter at the first and second flower truss, whereas it had no significant effect on the basal stem diameter (Supplementary Table S1). The final plant height was similar between the two VPD treatments. The leaf mass per area (LMA) was significantly reduced in the low-VPD treatment (Supplementary Table S1), indicating a thinner and looser leaf structure. The leaf area per plant in the low-VPD treatment was significantly higher than in plants grown in the high-VPD treatment approximately 20 days after transplanting (Supplementary Fig. S2).

**Figure 3:**
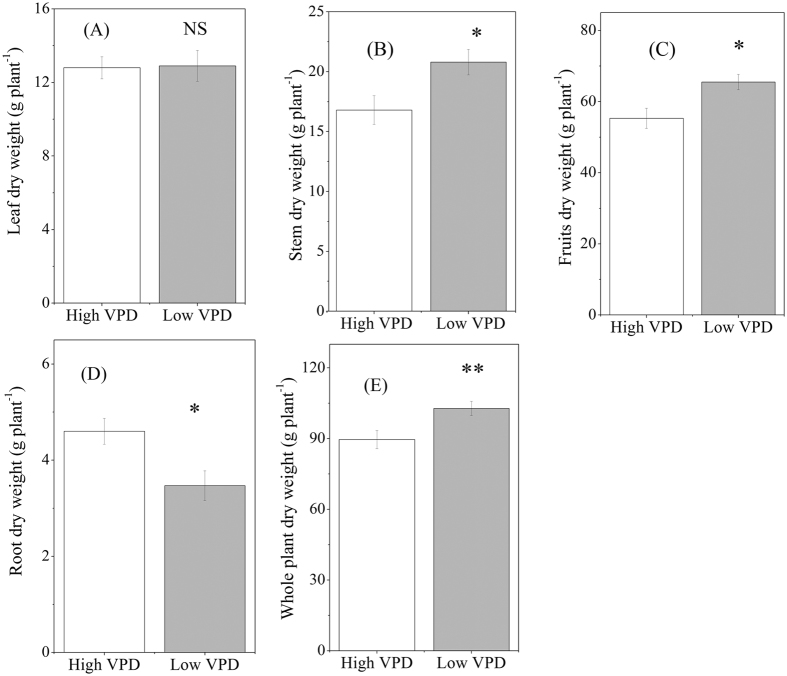
Effect of VPD regulation on the biomass production of leaf (A), stem (B), fruit (C), root (D) and whole plant (E).Values are means ± SE (n = 10). Significant differences between high- and low-VPD treatments were examined using Tukey’s test. *Significant at P < 0.05; **Significant at P < 0.01. NS: non-significant difference.

Reducing VPD by humidification significantly increased biomass production of the stem and fruits, whereas the leaf dry biomass was similar between the two treatments (Fig. 3A,B,C). In contrast to the enhancement in shoot growth, reducing VPD significantly decreased the root dry biomass (Fig. 3D). Taken together, the whole plant biomass was substantially increased by 27.3% in the low-VPD treatment (Fig. 3E).

**Figure 4:**
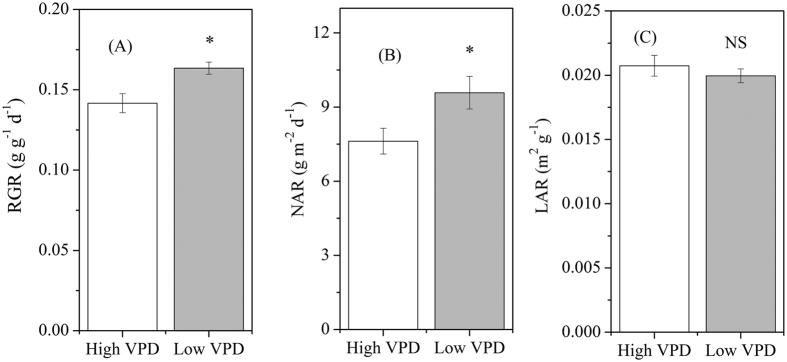
Effect of VPD regulation on plant growth parameters. RGR (relative growth rate, **A**) NAR (net assimilation rate, **B**) and LAR (leaf area ratio, **C**) were analysed in plants sampled at 0, 20 and 40 d after transplanting. Values are means ± SE (n = 10). Significant differences between high- and low-VPD treatments were examined using Tukey’s test. *Significant at P < 0.05; NS: non-significant difference.

The corresponding growth parameters in response to VPD regulation were analysed and shown in Fig. 4. Reducing VPD significantly increased the relative growth rate (RGR) and the net assimilation rate (NAR) compared with those in the high-VPD-grown plants, whereas the leaf area ratio (LAR) was similar between two treatments over the long-term acclimation (Fig. 4A,B,C).

The enhancement in the plant growth was accompanied by a substantial improvement in productivity: reducing VPD significantly increased the fruit yield per plant by 22.7% (Supplementary Fig. S3). In contrast, no significant differences in acidity and soluble solid content in fruits were revealed between the VPD treatments (Supplementary Fig. S3).

### VPD control in relation to water consumption and water use efficiency

**Figure 5:**
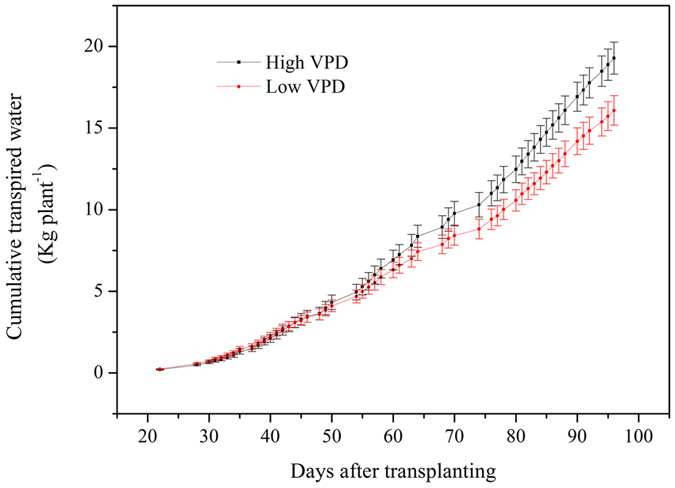
Effect of VPD regulation on the accumulation of plant-transpired water over experimental periods. Values are means ± SE (n = 10).

**Figure 6:**
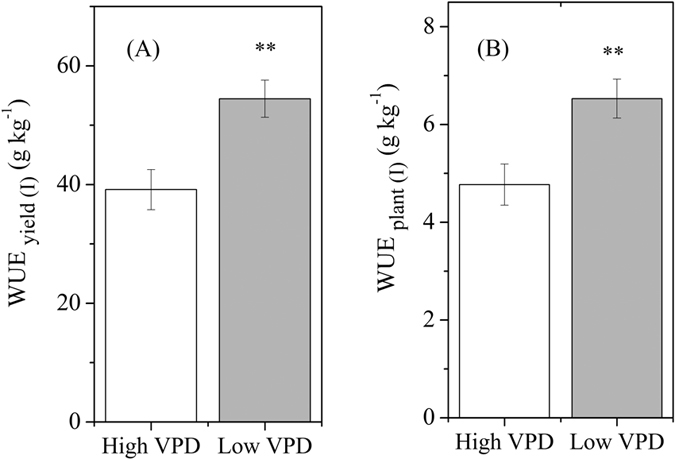
Effect of VPD regulation on irrigation water use efficiency of fruit yield (A) and plant biomass (B). WUE_yield(I)_, irrigation water use efficiency of fruit yield; WUE_plant(I)_, irrigation water use efficiency of plant biomass. Values are means ± SE (n = 10). Significant differences between high- and low-VPD treatments were examined using Tukey’s test. **Significant at P < 0.01.

VPD regulation had no significant effect on plant cumulative transpiration at the initial vegetative growth stage (Fig. 5). The reduction in cumulative transpired water by reducing VPD was enhanced with plant growth (Fig. 5). As a result, the cumulative transpired water consumption over the experimental period was significantly reduced by 19.9% per plant by reducing the VPD. In accordance with the principles of plant physiology, irrigation water use efficiency as criterion of plant biomass (WUE_plant-I_) and yield (WUE_yield-I_) in the low-VPD treatment were substantially improved by 36.8% and 39.1%, respectively (Fig. 6).

**Figure 7:**
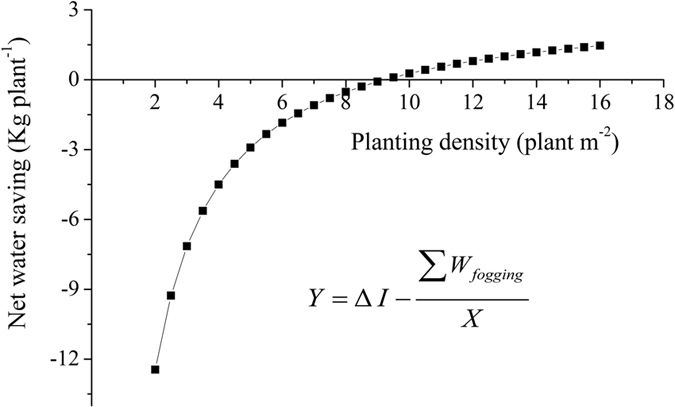
An inverse proportional function describing the relationship between net water saving and planting densities in fogging treatment. Where ∆I was saved irrigation by VPD regulation, kg plant^−1^; ΣW_fogging_ was fogging water consumption per unit of area, kg m^−2^. This model was parameterized based on assumption that plant growth and microclimate were uniformity in the application of fogging. Since daily water loss due to transpiration (T, kg palnt^−1^) was counterbalanced by adding exact amounts of irrigation in present study, thus the amount of irrigation was equal to cumulative transpired water consumption (∆T = ∆I). ∆I and ΣW_fogging_ were considered as constant parameters (3.4 kg plant^−1^ and 31.8 kg m^−2^, respectively), which was derived from experiments.

Input of water in greenhouse production systems consisted of irrigation and fogging water consumption. For the low-VPD treatment, the variation in weekly fogging water consumption was shown in Supplementary Fig. S4. The cumulative input of fogging water during the experimental period was 31.8 kg·m^−2^ in the cultivation area. Divided by planting density, the cumulative fogging water consumption per cultivation area can be transformed to individual plant consumption. In combination with the reduction in irrigation, the relationship between net water savings per plant and planting density can be described as an inverse proportional function (Fig. 7). This empirical function was developed on the assumption that plant growth and the microclimate were maintained uniformity, as described in detail in Supplementary Note 1. The net water savings for individual plants increased with planting density. The net water savings were positive at high planting densities (P ≥ 9 plant m^−2^) and negative at relative low planting densities (P < 9 plant m^−2^) (Fig. 7; Supplementary Note1). At relative low planting densities, the total input of water in the low-VPD treatment was significantly higher than in the high-VPD treatment (Supplementary Table S2). At high planting densities, the total input of water was similar between the low- and high-VPD treatments (Supplementary Table S2). Combined with the increase in whole-plant biomass and fruit yield, reducing VPD significantly increased WUE_plant(I+F)_ and WUE_yield(I+F)_ at high planting densities, whereas no significant effect was found at relative low planting densities (Supplementary Table S2).

## Discussion

In the present study, VPD regulation using a greenhouse fogging system effectively maintained the water balance and moderated water stress by reducing the evaporative driving force for water transport. However, VPD control had distinct implications for modulating water consumption and water use efficiency from physiological and system-wide perspective.

### VPD regulation moderated plant water stress and maintained water balance by reducing the evaporative driving force for water movement

The calculation of the CWSI confirmed that plants were less stressed by reducing the VPD. Based on physical principles, the midday greenhouse VPD contributed to excess negative pressures of the atmosphere. The atmospheric driving force was much larger than the water potential gradient between soil and leaf (Fig. 1) from a hydraulic perspective. The large difference in driving force between the soil-to-leaf system and the leaf-atmosphere interface can account for the disruption in water balance despite the plants being well irrigated. According to physical principles and experimental evidence, regulating the atmospheric moisture was an efficient solution for suppressing the excess driving force. The mass of plant water transport through the plant must balance the transpiration losses from the leaves, which can be expressed as follows^15^,^16^:


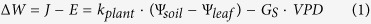


where W is the leaf water content and J and E are the rate of water supply and loss, respectively; Gs is the canopy stomatal conductance. If the water balance is to be maintained, J must equal E, and then ∆W = 0. Leaf dehydration was the foremost bottleneck caused by a large evaporative driving force, followed by disorders in plant water relations^17^,^18^,^19^. Leaf dehydration was alleviated in the low-VPD treatment by suppressing the evaporative driving force and reducing the transpiration rate. Consequently, a decline in leaf water potential was effectively prevented and a relative homeostatic water status was achieved in the low-VPD-grown plants. Along the soil-to-leaf pathway, the limitation in whole-plant hydraulic conductance was effectively moderated by reducing the VPD, implying a low resistance for water flow^20^. Previously, it has been suggested that plant hydraulic conductance must be sufficiently high to the sustain water supply and, consequently, that plant hydraulic properties strongly constrain leaf water status^21^,^22^,^23^. In combination with preventing the decline in leaf water potential, the turgor pressure of the guard cells was sufficiently high to sustain pore openness in low-VPD-grown plants. Taken together, present study showed that VPD regulation moderated plant water stress by alleviating leaf dehydration, preventing declines in leaf water potential and plant hydraulic conductance, and sustaining stomatal function.

### VPD regulation improved water use efficiency by reducing transpiration and improving photosynthesis in terms of plant physiological criteria

For the water transport process, the evaporative driving force was substantially reduced via VPD regulation, as described above. Consequently, suppression in driving force translated into a substantial reduction in water flow rate and cumulative transpired water consumption, which was confirmed by the present experimental evidence.

In addition to improving physiological water use efficiency by reducing water loss, enhancing carbon (biomass) production was also an efficient solution. The plant productivity processes of plant growth, biomass and yield production were improved by VPD regulation. Photosynthesis process was central to the improvement in biomass and yield production^24^. Even a small increase in the photosynthesis rate can translate into a large improvement in biomass and yield because the carbon gain extends over growing times and crop canopy^25^. A comprehensive quantification considering dynamic leaf expansion and biomass production suggested that the improvement in RGR was mainly determined by NAR. The growth analysis in combination with the gas exchange parameters highlighted the role of improved photosynthetic capacity in enhancing plant productivity.

The improvement in photosynthesis in low-VPD-grown plants can be partly attributed to the alleviation of stomatal closure. Stomatal limitation of CO_2_ uptake, the foremost bottleneck for the photosynthetic process, was efficiently mitigated by VPD regulation. The stomatal closure in response to plant water deficit in vascular plants is believed to be a passive process driven by reductions in the turgor pressure of the guard cells^26^. Because turgor pressure is directly linked to plant water status, the processes of water transport and loss can impact CO_2_ diffusion resistance by manipulating the physical and physiological characteristics of guard cells. As noted above, VPD regulation maintained the plant water balance by reducing the excessive driving force and flow rate. Combined with the less negative leaf water potential, the turgor pressure of guard cells was sufficiently high to sustain pore openness for CO_2_ utilization in the low-VPD-grown plants.

### Evaluation of net water savings and economical water productivity from a system-wide perspective incorporating input of fogging water

Water consumption in conventional agricultural production is generally estimated by the amount of irrigation or transpiration according to the discipline of plant physiology. In addition to irrigation, the fogging application for VPD regulation can account for the agricultural water consumption in greenhouse systems. In the present study, plant water consumption was evaluated on individual plant scales. The fogging water consumption was originally determined on the basis of cultivation area, which was translated to individual plant consumption according to planting densities. On individual plant scales, an empirical inverse proportional function was developed to describe the relationship between net water savings and planting density. The net water savings can be increased by increasing the planting density, which is an efficient solution for reducing fogging water consumption per plant. The reduction in irrigation was substantially counterbalanced by the fogging application, especially at low planting densities, as indicated by the negative net water savings. A high planting densities (P ≥ 9 plant m^−2^) was feasible and recommended to achieve positive net water saving in greenhouse systems, considering the trade-off between irrigation saving and input of fogging water. VPD regulation by fogging had a significant effect on improving agricultural water use efficiency at high planting densities (P ≥ 9 plant m^−2^), whereas VPD regulation had no significant effect on water use efficiency when fogging systems were applied for relatively low planting densities.

The reduction in irrigation per plant by VPD regulation was considered as a constant parameter in the theoretical analysis, and it was reliable when plants were cultivated at densities without sheltered canopies. When plants were cultivated without sheltered canopies, the planting density had relatively minor effects on the microclimate and individual plant transpiration. However, dense and sheltered canopies are inevitable at high planting densities, which would affect the microclimate and individual plant transpiration. Plant productivity was also affected by planting density. Previous research provided evidences that higher planting density increased total yield per area in greenhouse systems, but the yield of individual plants was reduced when planting density was too high^27^. In this case, transpiration and yield for individual plants would be density-varying variables. The optimal planting density for tomato varied, depending on varieties and agronomic practices. This research was conducted at a low planting density to avoid a dense and sheltered canopy; it is less certain how dense and sheltered canopies at high planting densities impact plant transpiration and productivity.

Improving the performance of the mechanical systems was an additional solution for improving the utilization efficiency and reducing input of fogging water. In addition to spraying water for environmental regulation, wasted water droplets on foliage or the ground surface also account for the consumption of fogging water to some extent. The improvement in evaporation efficiency allowed high proportions of fogging water to be directed towards environmental regulation and thus minimized fogging of non-evaporated water. The experimental fogging system in the present research was implemented with conventional nozzles, which generated large droplets and had a low fogging evaporate efficiency. There is considerable potential to improve the evaporation efficiency of the experimental fogging system. With increasing sophistication, nozzles were improved and designed for a micro-fog system, which dramatically reduced the droplet size^11^. Innovations in nozzles have facilitated a higher fog evaporation ratio, reducing the wetting of foliage and ground during application. A fogging operation in conjunction with fans or ventilation has been shown to improve evaporation efficiency^28^,^29^. These innovations are hypothesized to reduce fogging water consumption and enhance agricultural sustainability, but experimental evidence is needed.

## Conclusion

The present results demonstrated that atmospheric VPD played significant roles in modulating water movement along the soil-plant-atmospheric continuum, and these findings can be applied to greenhouse production. VPD regulation efficiently moderated plant water stress and maintained water balance by reducing the atmospheric driving force. From a physiological perspective, VPD regulation improved water use efficiency by reducing the consumption of transpired water and enhancing plant productivity. From a system-wide perspective, VPD regulation with present fogging system played a relatively minor role in reducing total water consumption in greenhouse systems. Although the suppression of the driving force by reducing VPD resulted in a substantial reduction in irrigation, the fogging application also accounted for the water consumption to some extent. The trade-off between reduction in irrigation and the input of fogging water can be optimized by increasing the planting density and improving the fogging evaporative efficiency.

## Materials and Methods

### Plant materials and growth conditions

The experimental site is an experimental station of Northwest Agriculture and Forestry University located in the Yangling demonstration zone, Shaanxi Province in northwest China (N34°15′, E108°04′, altitude 443.6 m). Experiments were conducted in two adjacent greenhouses with similar characteristics (15 m in length, 10 m in width and 3.5 m in height, north-south oriented) during the spring-summer season from March to June in 2016. The variability in air temperature, relative humidity and light intensity between the two greenhouses was examined prior to the experiments. Non-significant differences in these environmental factors were observed between two greenhouses. Tomato (*Solanum lycopersicum* L., CV. DiFen) seeds were sown in controlled-environment chambers for germination (22/18 °C for day/night temperature; white fluorescent lamps with 400 μmol m^−2^ s^−1^ PPFD). After four weeks, the seedlings (at four true-leaf stage) were transplanted to greenhouses at same time and grown in white-coloured pots (40 cm in diameter and 30 cm in height). The pots were filled with the same amount of garden mix substrate containing slow-release fertilizer. Apical shoots were pinched when the second flower truss started to bloom. Two trusses were retained for each plant with four fruits in each truss. The planting density was 3 plants m^−2^.

The soil surface was covered with a circular polythene sheet to prevent soil water evaporation. Plant transpiration was measured according to a standardized gravimetric approach of daily pot weighing with an electronic balance as described in a previous study^30^. Soil moisture was maintained uniformly at 85–90% field capacity. The daily water loss due to transpiration was counterbalanced by adding an exact amount of water to restore the moisture content to the desired target. Thus, the amount of irrigation was equal to cumulative transpired water consumption.

### Experimental design

Two experimental greenhouses were controlled under the same growth conditions but contrasting VPD: a midday VPD of 4–5 KPa was maintained under the natural greenhouse conditions without environmental regulation, serving as the high-VPD area; a midday VPD of 1–2 KPa was achieved by artificial humidification when the evaporative demand exceeded optimal ranges, serving as the low-VPD treatment. In the low-VPD compartment, the humidity was controlled using a fogging system (spray pressure: 2–6 MPa, droplet size: 25.8–66.2 μm) with a binary fluid mist nozzle. Spraying was automatically activated when the greenhouse VPD exceeded 1.2 KPa and turned off at the setpoint of 0.5 KPa considering the recommend values for tomato cultivation in greenhouses^31^,^32^. A randomized complete block design with five replications per treatment was adopted, resulting in a total of ten plots. There were ten plants per block.

### Environmental measurements

The air temperature (Ta), relative humidity (RH) and light intensity were observed by sensors (ZDR-20j, WuGe Instruments Co., Ltd., China) in the centre of each greenhouse installed at approximately 2.5 m above the ground. The VPD was calculated from the corresponding instantaneous air temperature and relative humidity. Data during the experimental periods were sampled every 10 minutes and recorded in a data-logger. All sensors were calibrated prior to the experiments.

### Measurements of leaf gas exchange

The leaf gas exchange parameters were measured with a portable photosynthesis system (LI-6400, Li-Cor, Inc., Lincoln, NE, USA) approximately 50 days after transplanting (9:00–12:00). All of the leaves were the youngest and fully expanded at the same plant nodes. During measurement, the environmental conditions in the leaf chamber were set close to those in the open-field condition in the greenhouse. The determination of gas exchange parameters was repeated with 10 plants in each treatment, and a total of three readings per plant were performed after steady state and equilibration. Two photosynthesis systems were used to enable simultaneous gas exchange measurements. The environmental conditions set for the measurements inside the leaf chambers were similar to the growth conditions in the greenhouse: leaf temperature, 35 ± 1 °C for the high VPD treatment and 30 ± 1 °C for low VPD treatment; light intensity, 1200 μmol m^−2^s^−1^, from red and blue light-emitting diodes; CO_2_ concentration, 400 ± 5 ppm; and air flow rate, 500 μmol s^−1^. The VPD was set at 3.2 KPa and 1.2 KPa for the high- and low-VPD-grown plants, respectively. The stomatal limitation (Ls) was estimated according to the following equation: Ls = 1 − C_i_/C_a_, where C_i_ is the intercellular CO_2_ concentration and C_a_ is the ambient CO_2_ concentration^33^. The intrinsic water use efficiency was calculated as the ratio between the photosynthesis rate (P_n_) and stomatal conductance (g_s_)^34^: intrinsic WUE = P_n_/g_s_.

### Determination of crop water stress index

The leaf temperature was determined at 10, 30 and 50 days after transplanting with a digital infrared thermometer (Model GM320) on seven healthy and mature leaves distributed randomly along the different layers of the canopy. The canopy temperature (T_c_) was calculated as the mean value of the measurements. The crop water stress index (CWSI) was calculated using the following equation^35^:


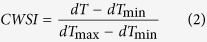


where dT is the difference between the canopy temperature (T_c_) and air temperature (T_a_): T_c_ − T_a_. dT_max_ is the upper limit of the canopy–air temperature difference that can be reached under the non-water-stressed condition. dT_min_ is the lower limit of the canopy–air temperature difference under the fully water-stressed condition. The values for the CWSI range from 0 to 1, where 0 indicates no stress and 1 indicates maximum stress. The upper and lower baselines of dT_max_ and dT_min_ were determined according to the relationship of the canopy–air temperature difference (T_c_ − T_a_) versus VPD under the non-water-stressed and fully-water-stressed condition, respectively, as previously described^35^. The baselines of dT_max_ and dT_min_ were estimated by independent experiments, as described in detail in Supplementary Fig. S5.

### Leaf water potential and relative water content

Following the determination of CWSI, leaves were cut and measured for fresh weight. To determine the turgid weight, the leaves were kept in distilled water in darkness until they reached a constant weight (full turgor, after 24 h). The relative water content (RWC) was calculated according to the equation RWC = (Fresh weight − Dry weight)/(Turgid weight − Dry weight). The leaf water potential (Ψ_leaf_) was immediately measured after cutting using a pressure chamber (PMS-1000, Corvallis, OR, USA). Measurement of the pre-dawn leaf water potential (predawn Ψ_leaf_) began at approximately 0430 h and finished prior to sunrise. The midday leaf water potential (midday Ψ_leaf_) was generally measured between 1230 h and 1330 h.

### Plant hydraulic conductance

The whole-plant hydraulic conductance (K_plant_) was simultaneously estimated with measurements of plant-water parameters according to the evaporative flux method^36^,^37^,^38^. K_plant_ was estimated according to the whole-plant transpiration rate (T_plant_) and the water potential drop between soil and leaf (Ψ_soil_-Ψ_leaf_) as described in the following equation:


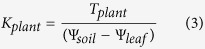


where Ψ_soil_ is the average soil water potential, which was assumed to equal the predawn Ψ_leaf_ because Ψ_soil_ remained relatively constant and reached equilibrium with the canopy water potential^39^,^40^. The plant transpiration rate (T_plant_) was estimated from the weight of the water loss from plants, as described by previous research^36^.

### Determination of plant growth and morphological parameters

The plant samples were homogenized for morphological criteria at the beginning of the experiment. The plants were sampled for biomass and leaf area measurements at 0, 20, 40, 70 and 90 days after transplanting. The leaf area per plant was measured using a Li-3000 leaf area meter (Li-Cor, Inc., Lincoln, Nebraska, USA). Samples were dried at 80 °C to constant weight and weighed. The final morphological parameters were determined after fruits harvest (about 90 days after transplanting).

The growth analysis parameters, including relative growth rate (RGR), net assimilation rate (NAR) and leaf area ratio (LAR), were calculated from the total dry weight and leaf area using the following equations^41^:


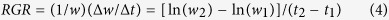


where W_1_ and W_2_ are the biomass of the whole plant at times t_1_ and t_2_,


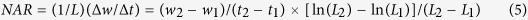


where L_1_ and L_2_ are the total leaf areas of the whole plants at times t_1_ and t_2_, and


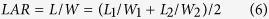


### Fruit characteristics and water use efficiency

The soluble solids content and acidity content were determined using a digital refractometer (PAL-BX/ACID3, Atago Co. Ltd., Tokyo, Japan) with automatic temperature compensation. The cumulative transpired water was estimated from the sum of the daily transpiration. The cumulative fogging water for VPD regulation was estimated from the sum of the daily amount, which was recorded by a flowmeter. Some non-evaporated water droplets were captured and recycled into the fogging system, and this volume was subtracted from the calculation of the total fogging water consumption. According to the principles of plant physiology, the whole-plant water use efficiency was calculated as the ratio of plant biomass (root and shoot) to cumulative irrigation^30^, referred to as WUE_plant (I)_. The fruit yield water use efficiency was calculated as the ratio between the fruit yield (grams of fruit) and the cumulative irrigation^42^, referred to as WUE_yield (I)_.

From a system-wide perspective, the total water consumption was defined as the sum of the cumulative water consumed by irrigation and fogging. The water use efficiency of the plant biomass and fruit yield were simultaneously evaluated based on the criterion of total water consumption, referred to as WUE_plant (I+F)_ and WUE_yield (I+F)_, respectively.

## Additional Information

**How to cite this article:** Zhang, D. *et al*. Vapour pressure deficit control in relation to water transport and water productivity in greenhouse tomato production during summer. *Sci. Rep.* **7**, 43461; doi: 10.1038/srep43461 (2017).

**Publisher's note:** Springer Nature remains neutral with regard to jurisdictional claims in published maps and institutional affiliations.

## Supplementary Material

Supplementary Information

## Figures and Tables

**Table 1:** Comparison of plant water status between high- and low-VPD treatments.

Traits	Units	High VPD	Low VPD	P
Predawn Ψ leaf	MPa	−0.262 ± 0.036	−0.233 ± 0.033	NS
Midday Ψ leaf	MPa	−1.47 ± 0.035	−1.03 ± 0.038	**
Midday K_plant_	mmol m^−2^ S^−1^ MPa^−1^	8.73 ± 0.703	11.8 ± 0.822	**
Midday T_canopy_	°C	33.7 ± 0.603	31.3 ± 0.325	**
Midday CWSI		0.367 ± 0.005	0.272 ± 0.008	*
Midday RWC		78.2% ± 0.911%	84.6% ± 1.70%	**

The data represent the means ± SE (n = 10). Ψ_leaf_, leaf water potential; K_plant,_ plant hydraulic conductance; T_canopy,_ canopy temperature; CWSI, crop water stress index; RWC, leaf relative water content. Significant differences between high- and low-VPD treatments were compared using Tukey’s test.*Significant at P < 0.05; **Significant at P < 0.01; NS: non-significant difference.
