# Supplementary material for: Vapour pressure deficit control in relation to water transport and water productivity in greenhouse tomato production during summer
Source: Sci Rep. 2017 Mar 7;7:43461. doi: 10.1038/srep43461 (PMC5339896; doi:10.1038/srep43461)
Supplement: Supplementary Information [file srep43461-s1.pdf]

Supplemental Information File

Vapour pressure deficit control in relation to water transport and water productivity in greenhouse tomato production during summer

Dalong Zhang, Qingjie Du, Zhi Zhang, Xiaocong Jiao, Xiaoming Song, Jianming Li*

Supplementary Table 1. Effect of VPD regulation on plant morphological parameters. LMA, leaf mass per area. Data represent means ±SE (n=10). Significant differences between high and low VPD treatments were compared using Tukey's test.* Significant at $P<0.05$;** Significant at $P<0.01$; NS: not significant.

Traits	Position	High VPD	Low VPD	P
Final leaf length (cm)	above first truss	31.8±0.898	34.1±0.660	*
	above second truss	31.2±1.02	38.6±1.78	**
Maximum leaf expansion rate ($cm\ d^{-1}$)	above first truss	1.43±0.031	1.62±0.031	**
	above second truss	1.48±0.037	1.85±0.046	**
Final stem diameter (mm)	basal	8.62±0.382	8.90±0.341	NS
	below first truss	9.76±0.359	10.8±0.391	*
	below second truss	9.02±0.393	10.1±0.293	*
Maximum stem expansion rate ($mm\ d^{-1}$)	basal	0.568±0.146	0.587±0.125	NS
	below first truss	0.532±0.024	0.641±0.021	**
	below second truss	0.445±0.021	0.589±0.020	**
Final plant height (cm)		94.3±1.44	96.6±3.31	NS
LMA ($g\ m^{-2}$)		52.1±2.27	37.2±3.12	**

Supplementary Table2. Comparison of water consumption and water productivity between high VPD and low VPD treatment, based on theoretical analysis. Parameters were determined on scale of individual plant. Where $W_{fogging}$ was fogging water consumption for individual plants, kg $plant^{-1}$; $W_{(I+F)}$ was total input of water for individual plants, including irrigation and fogging, kg $plant^{-1}$; Net $water_{saving}$ was net water saving by VPD regulation, kg $plant^{-1}$; $WUE_{plant\ (I+F)}$ was water use efficiency on criterion of whole plant biomass and total input of water, g kg^{-1}; $WUE_{yield\ (I+F)}$ was water use efficiency on criterion of fruits yield and total input of water, g kg^{-1}.

Planting density	4 plant m^{-2}			8 plant m^{-2}			12 plant m^{-2}		
	**High VPD**	**Low VPD**	**Sig**	**High VPD**	**Low VPD**	**Sig**	**High VPD**	**Low VPD**	**Sig**
$W_{fogging}$	0.00	7.95		0.00	3.98		0.00	2.65	
$W_{(I+F)}$	19.28 ± 0.61	24.03 ±0.80	**	19.28 ± 0.61	20.05±0.80	NS	19.28 ± 0.61	18.73±0.80	NS
Net $water_{saving}$	0.00	-4.75 ±0.62	**	0.00	-0.77±0.62	NS	0.00	0.55±0.62	NS
WUE plant $_{(I+F)}$	4.71 ±0.32	4.32 ± 0.20	NS	4.71 ± 0.32	5.21 ± 0.27	NS	4.71 ±0.32	5.63 ± 0.28	*
WUE yield $_{(I+F)}$	38.5 ± 1.89	36.0 ± 1.59	NS	38.5 ± 1.89	43.3 ± 2.14	NS	38.5 ± 1.89	46.5 ± 2.39	*

Supplementary Figure 1.Comparison of typical diurnal variation of greenhouse environmental factors including air temperature (A), relative humidity (B), VPD (C) and light intensity (D) in the high and low VPD compartments on a sunny day(13 June 2016).

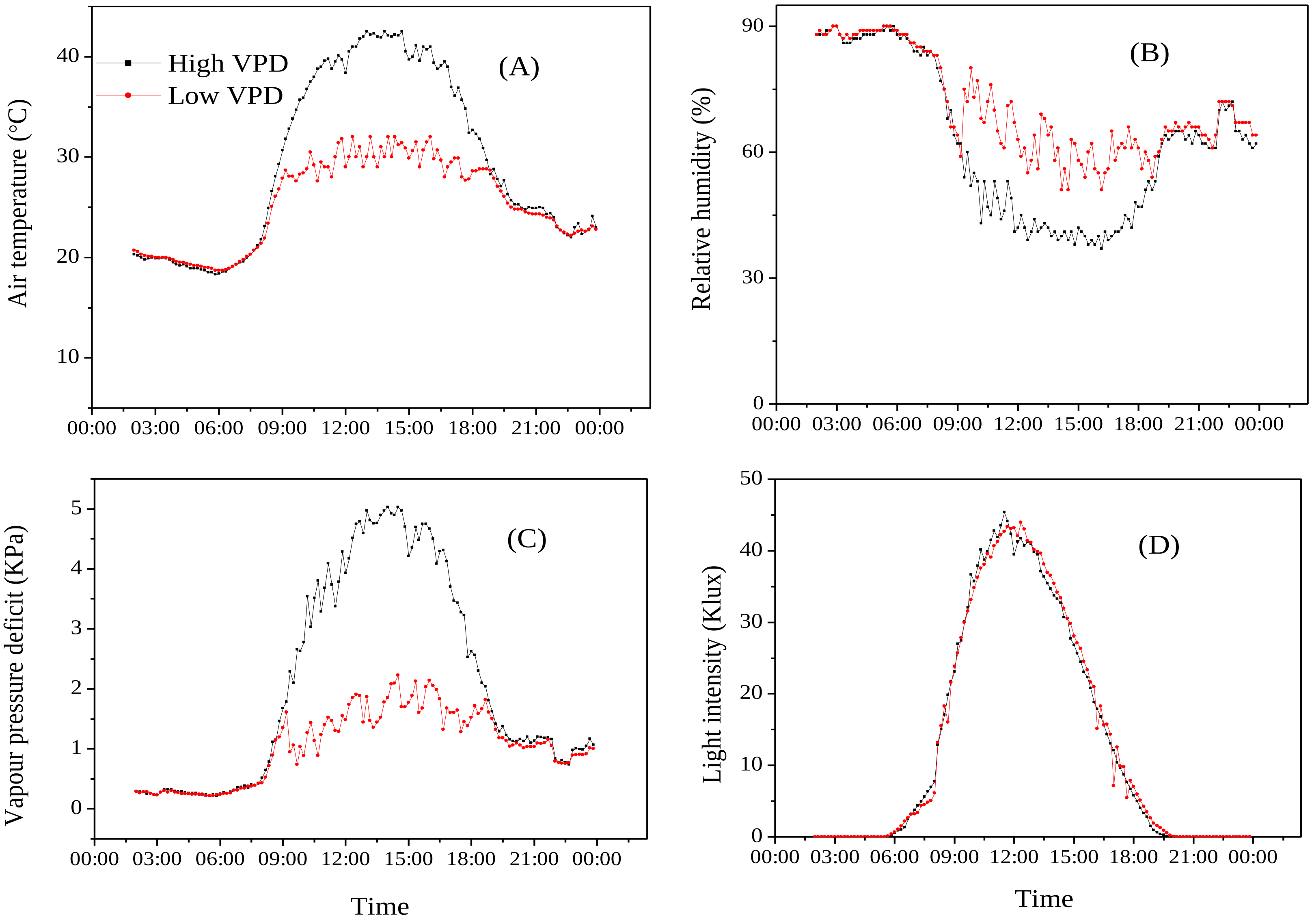

Supplementary Figure 2.Effect of humidification on leaf area. Data represent means ±SE (n=10). Significant difference between high and low VPD treatments were compared using Tukey's test.* Significant at P<0.05;** Significant at P<0.01; NS: not significant.

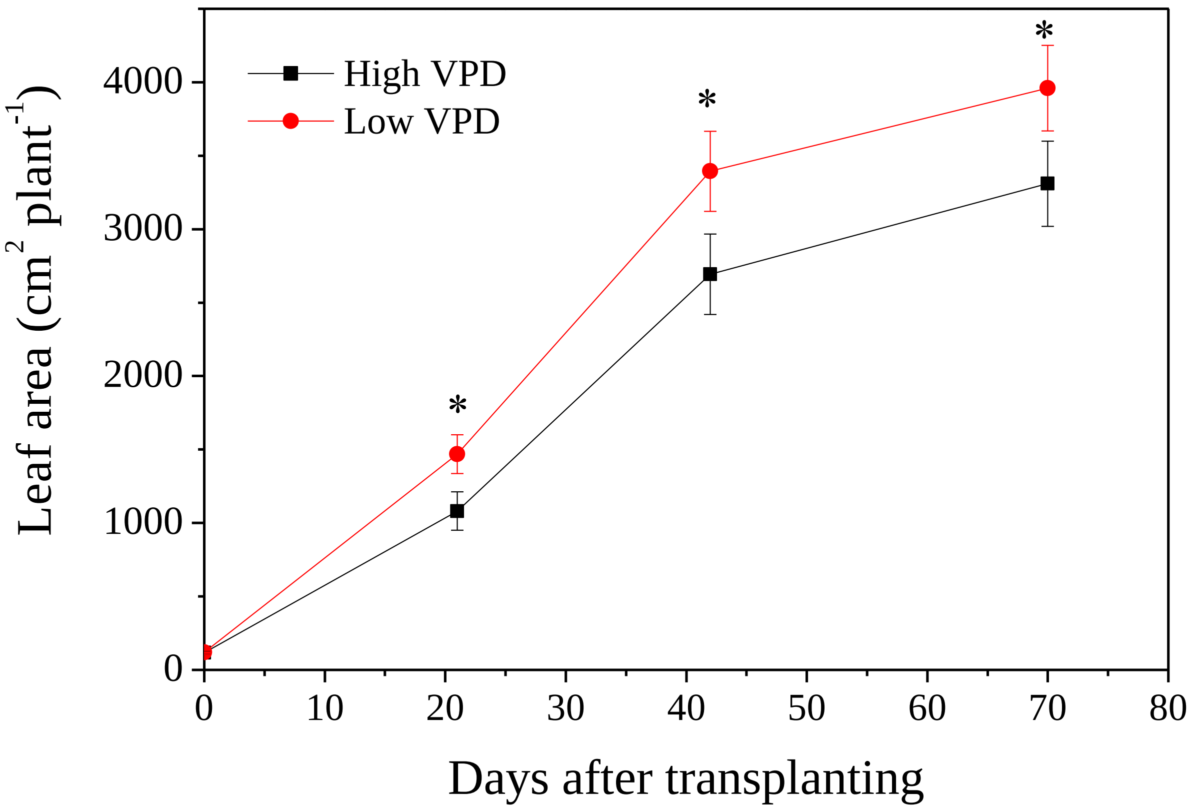

Supplementary Figure3. Effect of VPD regulation on fruit yield and fruit quality. Values are means ± SE (n=22). Significant differences between high- and low-VPD treatments were examined using Tukey's test. ** Significant at $P<0.01$. NS: non-significant difference.

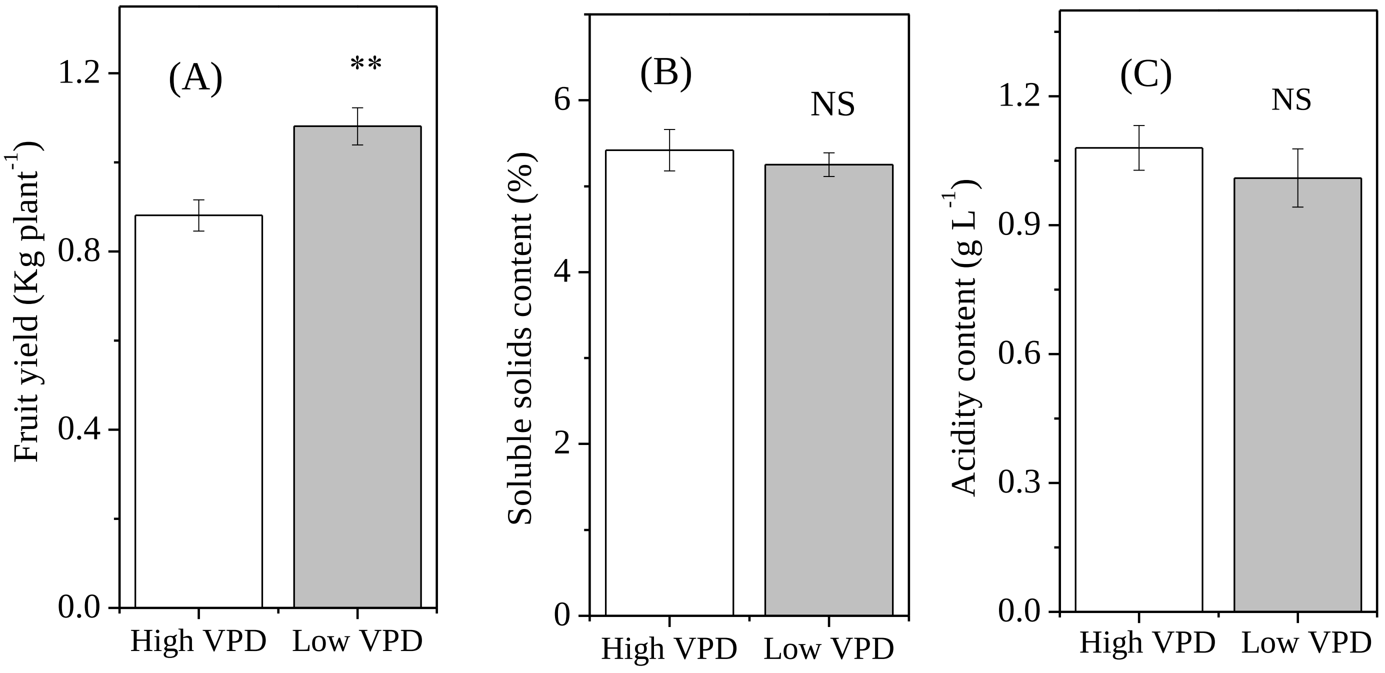

Supplementary Figure4 .Weekly variation of fogging water consumption for VPD regulation.

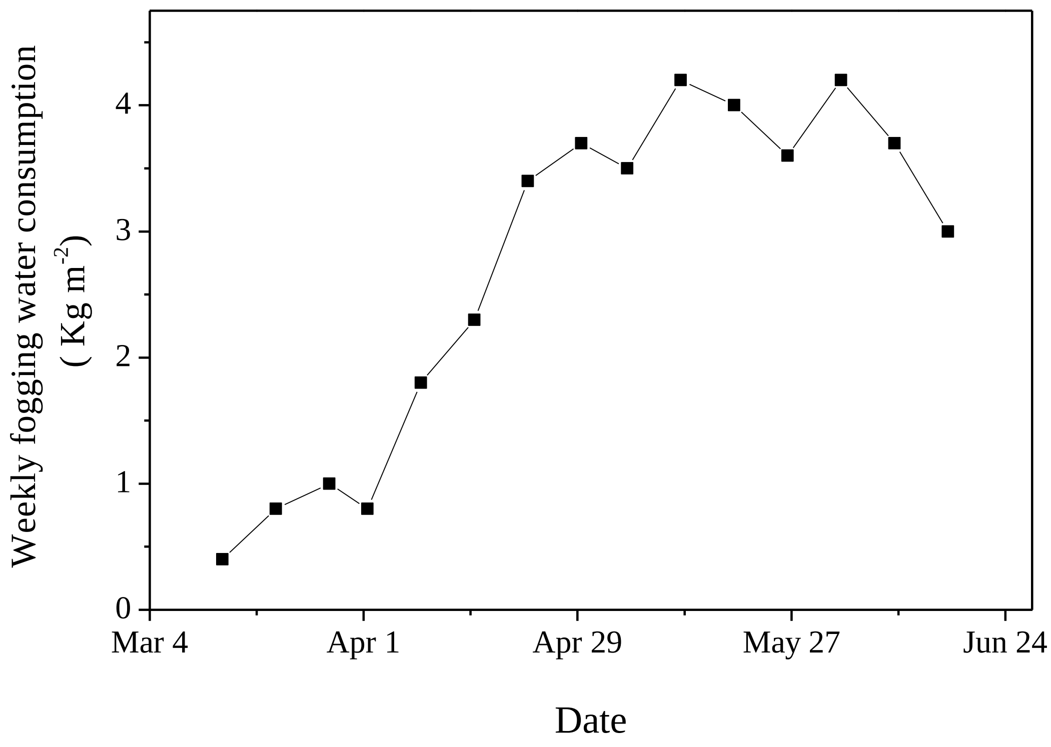

Supplementary Figure5.Relationship between canopy–air temperature difference (T c-T a) and VPD for well watered and maximally stressed tomato. A was the point which was used as an example of how CWSI value was calculated. B and C represent upper and lower limits for A, which was determined under non-water-stressed and fully-water-stressed condition, respectively. BC was the vertical distance between lower and upper baseline. AC was vertical distance between lower baseline and point A. CWSI was crop water stress index, which was determined as : CWSI= AC/BC.

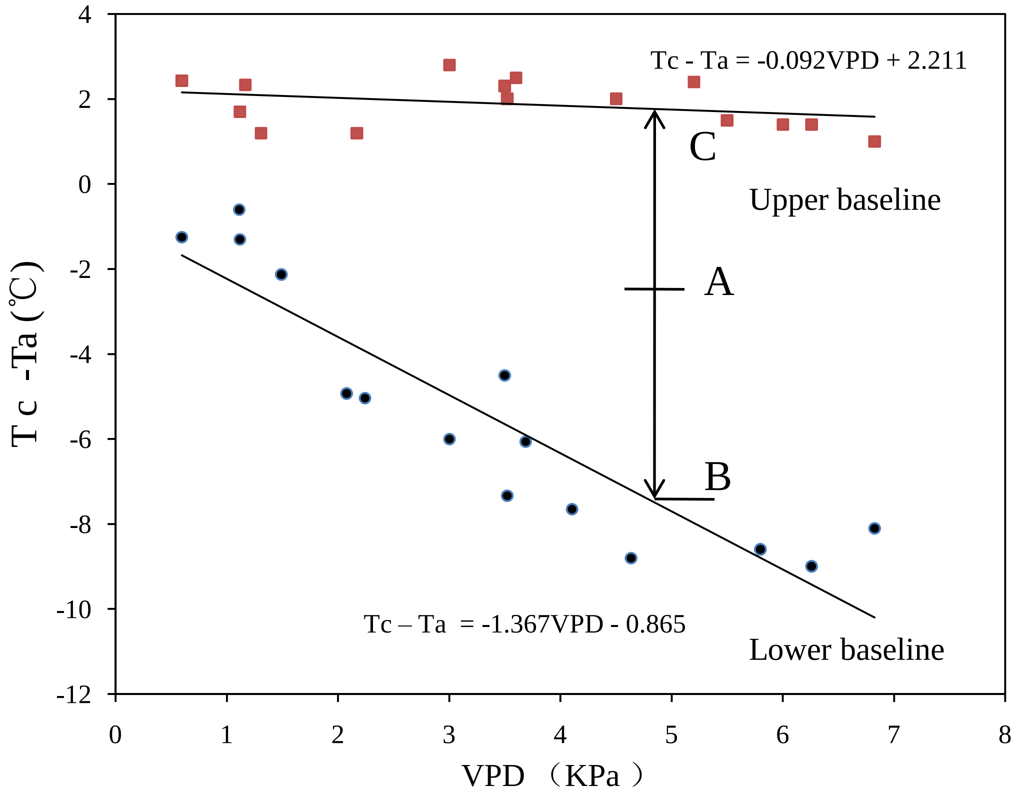

Supplementary note1.Theoretical analysis of the relationship between net water saving and planting density

Net water saving (net W_{saving}, Kg palnt^{-1}) was determined according to difference in total input of water between high VPD treatment ($W_{high\ VPD}$, Kg palnt^{-1}) and low VPD treatment ($W_{low\ VPD}$, Kg palnt^{-1}):

$$\text{net } W_{saving} = W_{high\ VPD} - W_{low\ VPD} \quad (1)$$

For high VPD treatment, input of water in greenhouse systems was solely applied for irrigation ($I_{high\ VPD}$, Kg palnt^{-1}); for low VPD treatment, input of water in greenhouse systems consisted of irrigation ($I_{low\ VPD}$, Kg palnt^{-1}) and fogging water consumption ($W_{fogging}$, Kg palnt^{-1}). Thus, equation (1) can be expressed as the following equation:

$$\text{net } W_{saving} = I_{high\ VPD} - (I_{low\ VPD} + W_{fogging}) = \Delta I - W_{fogging} \quad (2)$$

Where ΔI was saved irrigation by VPD regulation (Kg plant^{-1}). Since daily water loss due to transpiration was counterbalanced by adding exact amounts of irrigation in present study, thus the amount of irrigation was equal to cumulative transpired water consumption (T, Kg palnt^{-1}). Equation (2) can be expressed as:

$$\text{net } W_{saving} = \Delta I - W_{fogging} = \Delta T - W_{fogging} \quad (3)$$

Fogging water consumption for individual plants was determined by total fogging water consumption and the number of plants within a given cultivation area. Thus, fogging water consumption for individual plants can be estimated according to input of fogging water ($\Sigma W_{fogging}$, kg m^{-2}) per unit of area and planting density (P, n m^{-2}) :

$$W_{fogging} = \Sigma W_{fogging}/P \quad (4)$$

Net water saving per plant can be determined by substituting equation (4) in equation (3),

$$netW_{saving} = \Delta T - \frac{\sum W_{fogging}}{P} \quad (4)$$

The theoretical threshold value of planting density determining positive and negative net water saving can be estimated according to equation (4):

$$P > \frac{W_{fogging}}{T_{saving}} \approx 9$$
